# Optimum drying conditions for ginger (*Zingiber officinale* Roscoe) based on time, energy consumption and physicochemical quality

**DOI:** 10.1016/j.fochx.2023.100987

**Published:** 2023-11-10

**Authors:** Kaikang Chen, Yanwei Yuan, Bo Zhao, Mohammad Kaveh, Mohsen Beigi, Yongjun Zheng, Mehdi Torki

**Affiliations:** a College of Engineering, China Agricultural University, Beijing 100089, China; b National Key Laboratory of Agricultural Equipment Technology, Chinese Academy of Agricultural Mechanization Sciences, Beijing 100083 China; c Department of Petroleum Engineering, Collage of Engineering, Knowledge University, 44001 Erbil, Iraq; d Department of Mechanical Engineering, Tiran Branch, Islamic Azad University, Tiran, Iran; e Department of Computer Engineering, Faculty of Electrical and Computer Engineering, Technical and Vocational University (TVU), Tehran, Iran

**Keywords:** Sonication, Ginger, Energy consumption, Quality attributes, Solid-phase microextraction, Optimized drying

## Abstract

The effect of ultrasonic pre-treatment on moisture removal characteristics of ginger in a convective dryer was investigated. The slabs were dried by practicing sonication durations of 0, 15 and 30 min at different levels of the air temperature and velocity. Following increasing the sonication duration and air temperature, required time and energy to dehydrate the samples were decreased. The pre-treatment played important role in improving rehydration capability and surface color retention in the dried gingers. Content of the main volatile component (α-Zingiberene) was not influenced by the sonication. Mean values for the phenolic contents and antioxidant activity at sonication duration of 0, 15 and 30 min were determined to be 18.93, 18.15 and 17.49 GAE/g dry matter and 83.57, 78.33 and 74.58 %, respectively. The desired values for the temperature, velocity and sonication duration were revealed to be about 66 °C, 3 m/s and 20 min, respectively.

## Highlights

• Ultrasonic pre-treatment was applied before hot air drying of ginger.
• Influence of sonication time and the air temperature and velocity was studied.
• Sonication led to the shorter process duration and lower energy consumption.
• The pre-treatment resulted in better physical quality and more extract yield.
• Drying temperature had the main impact on the studied parameters.

## 1 Introduction

Significant amounts of agricultural products are wasted mainly due to their high moisture contents. Therefore, producing dried products can compensate for a major part of the food lack in the world while preventing the wastage of these materials. In other words, drying of agricultural and food products could be the missing link in the global food security chain. Additionally, due to the change in people's taste and desire to consume diverse products, drying of the foodstuffs has recently received more attention.

However, the drying process is a complex process that usually requires a lot of energy and causes important changes in foodstuffs’ characteristics (physical, chemical and nutritional) (Zhu et al., 2023). Hence, it is necessary to do this process properly. By emphasizing on shorter, cleaner and higher-quality drying of the products, researchers and industry owners try to develop more effective drying systems. To achieve to these targets, favourable competence of the specific novel drying technologies such as pulsed electric field, low-pressure superheated steam, microwave heating, and radio frequency have been recently reported (Wang et al., 2023). However, due to serious limitations such as high price of use, need for continuous control of the process and inadequate consumer acceptance, their industrial implementation faces serious obstacles.

Force hot air is a commonly practiced method to dry a wide range of different products since it is cost-effective and easy to use (Beigi, 2019). This techniqaue is almost the first idea to develop industrial dryers. The main functional mechanism of a typical convective dryer is simultaneous transfer of warmth and moisture. Despite the unique benefits, mainly because of great heat needed to vaporize the water from the object and low thermal conductivity, hot air dryers use energy in an inefficient way and usually destroy the final product quality due to the long term processing (Geng et al., 2023).

Utilization of additional (hybrid) energy sources and pre-treatment of the subjected product prior to drying process are the two main solutions investigated to increase effectiveness of the hot air drying systems (Ghanbarian et al., 2020). Because of non-thermal quiddity with mechanical effects, utilization of ultrasonic power has recently gained increasing in food industry. To improve drying characteristics of different foodstuffs, the power has been practiced in the both ways (pre-treatment and hybrid) (Tao et al., 2021). In case of pre-treatment, according to the reported observations in the open literature, it could be generally stated that ultrasonic pre-treatment causes the drying process shorter and results in a certain lower energy consumption in a hot air dryer. However, it is difficult to make a definitive conclusion about how it affects the foodstuffs characteristics since the qualitative traits generally are affected by different factors mainly including the product nature and drying conditions. Therefore, here, ultrasonic sonication was implemented before hot air drying of ginger slices and, in addition to the time and energy consumption, the main quality features of the dried ginger were assessed. The most desirable drying conditions were also tried to be identified by using response surface method (RSM).

## 2 Material and methods

### 2.1 Fresh gingers

The fully matured fresh gingers (*Zingiber officinale* Roscoe) were harvested from a research greenhouse in September 2022. Before the experiments, the gingers were stored in sand in a dark room at controlled temperature of 15 °C and relative humidity of 50 % (Ding et al., 2012). Practicing the standard vacuum oven procedure at temperature of 50 °C and pressure of 13.3 kPa (An et al., 2016), moisture in the fresh gingers was m to be 0.81 ± 0.12 (wet basis).

### 2.2 Ultrasonic pre-treatment and drying experiments

To use in each drying experiment, nearly 400 g of the fresh gingers was washed, peeled and sliced into 4 ± 0.5 mm slabs by a cutting machine. For sonication the ginger slices prior to drying experiments, an ultrasonic bath (Farasot Zagros Co., Iran, 5 L, 40 kHz, 200 W) was used. The samples: water ratio was adjusted to be 1:5, and the water temperature controlled to be about 25 °C. Three duration levels (0, 15 and 30 min) for the pre-treatment were selected. Drying of the gingers was carried out in a laboratory hot air system by practicing the air temperatures of 55, 65 and 75 °C and flow rates of 1, 2 and 3 m/s. To dry the gingers, the prepared slabs were spread on the drying tray as a monolayer. The plot mass was continuously monitored by a balance (ViBRA, EG 620-3NM, Japan), and moisture content of the gingers at each weighing time determined using Eq. (1) (Ghanbarian et al., 2020):

(1) $$M=1-\frac{\left({1-M}_{0}\right)\times m_{0}}{m}$$

In Eq. (1), *M* and *m* are the product moisture content (wet basis) and mass (kg), respectively. Also, *0* denotes to the initial condition.

Drying of the gingers was continued till the samples’ moisture content reached about 0.12 (wet basis). Furthermore, a power meter (Ziegler instruments Co., Germany) was used to measure the total consumed energy; and required energy (MJ) for removing 1 kg water from the samples (specific energy consumption, *SEC*).

### 2.3 Rehydration ratio

Three dehydrated ginger slabs were weighed and soaked for 30 min into a 300 mL distilled water in a lab water bath (Parmis Teb Azma Co., Iran). During the rehydration period, temperature of the water was controlled and adjusted to be constant at 30 °C. After completing the process, the slices were taken out, drained for 2 min over a mesh, wiped by using absorbent paper, and weighed. Finally, water absorption capacity was determined based on the formula proposed by Xu et al. (2021).

### 2.4 Total surface color change

To measure color of the samples’ surface, the CIE lab system with a digital camera was used to provide surface images from the randomly selected fresh and dried gingers. The color indices (L, a and b) for the samples’ surface were determined according to the methodology described by Nadian et al. (2015), and total change in the color (*ΔE*) was computed as follow (Wang et al., 2021):

(2) $$\Delta E={\left[{\left(L_{f}-L_{d}\right)}^{2}+{\left(a_{f}-a_{d}\right)}^{2}+{\left(b_{f}-b_{d}\right)}^{2}\right]}^{0.5}$$

In Eq. (4), *f* and *d* indicate the fresh and dried, respectively.

### 2.5 Analysis of volatile components

To analysis the volatile composition, extract preparation from the fresh and processed samples was done based on the solid-phase microextraction methodology described by An et al. (2016) and Ding et al. (2012). To this end, the fresh and dried samples were blended and milled at lab temperature (20–25 °C), and the obtained powders were sieved by using a wire screen (80 mesh). For each experiment, accurately weighed 1000 mg of the samples was placed in a glass vial and sealed. To achieve a saturated solution, further 400 mg sodium chloride was added to the homogenized pastes and mixed completely. All the vials were stowed at the lab temperature till extraction started. Finally, solid-phase micro-extraction included a polydimethyl-siloxane fiber column with 100 μm length was used to isolate the volatile components. The column was implanted for 30 min into the headspace at 40 °C, and eluted for 5 min at 250 °C. Identification of chemical constituents of the extracts was conducted by GC/MS analysis based the methodology descrisbed by Ding et al., 2012.

### 2.6 Phenolic contents and antioxidant activity

To prepare methanol extract of the samples, the procedure described Ekramian et al. (2021) was accurately implemented. The phenolic contents and antioxidant activity in the methanol extracts were exactly determined following the Folin-Ciocalteu procedure proposed by Chan et al. (2009) and DPPH methodology described by An et al. (2016), respectively.

(3) $$AA\left(\%\right)=\left(\frac{{\mathrm{Abs}}_{\mathrm{control}}{-Abs}_{\mathrm{sample}}}{{\mathrm{Abs}}_{\mathrm{control}}}\right)\times 100$$

### 2.7 Optimization drying variables

The well-known RSM/Box-Behnken design approach was implemented to determine the optimum drying inputs including the air temperature (T) and flow rate (V) and sonication duration (SD). The process time, *SEC*, RR, *ΔE*, extract yield, TPC, AA, and the main volatile component - which was identified through the analysis- were evaluated as the important parameters for the ginger drying. In the present research work, the aim of optimization was to achieve minimized process time, *SEC* and *ΔE* as well as maximized RR, extract yield, TPC, AA, and the main volatile component. Optimization was completed based on the second order polynomial function.

Furthermore, total desirability of the model (*D*) was computed as follow:

(4) $$D={\left(d_{i}\times d_{\mathrm{ii}}\times \cdots \times d_{n}\right)}^{1/n}$$

In Eq. (4), *d* indicates desirability of each variable, and *n* is number of the variables.

### 2.8 Statistical analysis

In this study, SPSS (19.0) and ANOVA procedure were used to assess the impact of the pre-treatment duration and drying air variables on the considered indices. Duncan’s test was also used to compare the means.

## 3 Results and discussion

### 3.1 Process duration

The mean values of the process duration are listed in Table 1. In addition, to validate the experimental repeatability, relative standard deviation (RSD) values were analyzed and the outcomes are represented in the table. As shown, average drying time of the samples changed from 132 to 523 min. The RSDs were in the range of 1.51–8.43 % (mean value of 4.28 %) indicating that, data repeatability of the process time has been excellently succeeded. Experimental data repeatability for drying process duration is usually influenced by the drying conditions as well as the characteristics of the samples. According to the findings obtained for the time repeatability, it could be concluded that not only the drying conditions have been controlled and adjusted properly throughout the experiments but also the ginger samples prepared with a high accuracy manner.

**Table 1:** The process duration and specific energy consumption (*SEC*) for ginger drying at the operating conditions.

T (°C)	V (m/s)	SD (min)	Time (min)		*SEC* (MJ/kg)	
			Mean	Relative standard deviation (%)	Mean	Relative standard deviation (%)
55	1	0	523	2.29	29.81	4.53
		15	475	3.58	27.53	7.30
		30	439	2.05	25.92	6.33
	2	0	489	2.66	32.64	6.00
		15	452	3.32	30.62	6.89
		30	416	1.68	28.67	4.33
	3	0	468	2.35	36.15	4.40
		15	440	2.05	34.44	3.95
		30	397	1.51	31.57	3.52
65	1	0	348	2.30	23.49	7.41
		15	309	3.56	21.31	9.06
		30	291	3.44	20.54	8.81
	2	0	321	5.30	29.61	7.40
		15	293	5.12	27.48	7.46
		30	275	4.73	26.27	6.20
	3	0	306	5.23	28.92	7.33
		15	271	2.58	26.06	3.99
		30	260	2.31	25.47	2.63
75	1	0	193	7.25	16.07	11.01
		15	168	3.57	14.44	6.65
		30	151	7.28	13.47	11.06
	2	0	178	8.43	20.56	9.05
		15	155	8.39	18.35	8.77
		30	143	4.90	17.42	7.41
	3	0	168	5.95	23.31	5.79
		15	145	7.59	20.57	7.34
		30	132	6.06	19.22	7.28

From the experimental results and based on the ANOVA, at constant levels of the drying air velocity and sonication duration, the temperature increasing from 55 to 65 and from 65 to 75 °C caused noteworthy (*P < 0.05*) reduction in time required to dehydrate the samples. At higher drying temperature, both the facilitated diffusion of moisture inside the object and moisture evaporation from the product surface generally occur resulting in shortened the process duration. Several researches have confirmed faster drying at the higher temperatures for different crops such as wormwood leaves (Beigi, 2018), cumin seeds (Namjoo et al., 2022) and peppermint leaves (Ghanbarian et al., 2020).

The observations obtained through the experiments discovered that higher flow rates of the drying air shortened the process. The same finding has been reported by several researchers such as Akpinar and Toraman (2016) and Tohidi et al. (2017). The influence of the velocity is mainly due to the reduced boundary layer resistance at the higher air low rates. Detailed discussion for this topic could be found in the reported study by Tohidi et al. (2017).

From Table 1 and the performed statistical analysis, a considerable (*P < 0.05*) reduction in the process duration could be observed by applying the ultrasonic pre-treatment. The finding is well confirmed with Cao et al. (2018) for barley grass before freeze drying, Rani and Tripathy (2019) for pineapples dried by hot air and Bassey et al. (2022) for infrared drying of red dragons. As stated by Rani and Tripathy (2019), sonication (acoustic cavitation) prior to drying process forms a grid of micro channels inside the biological products due to the sponge effect. The phenomena helps to enhance the water diffusion and facilitate moisture elimination from the samples, and accordingly reduces the process duration.

### 3.2 Energy consumption

From Table 1, the energy needed to eliminate 1 kg water of the ginger samples ranged from 13.47 to 36.15 MJ, where was augmented at the higher air velocities and reduced by any augmentation in the air temperature and sonication duration as well. From statistical analysis, the mean values for the consumed energy at the temperatures of 55, 65 and 75 °C was determined to be 30.82, 25.46 and 18.16 MJ/kg, where differences among the values were important (*P < 0.05*). In case of drying air temperature, shortened process duration is the key rationale for the lower energy consumption. However, although the higher flow rates resulted in faster moisture removal from the ginger samples but generally the consumed energy was increased due to higher volumetric flow rate and shorter interaction phase between the air stream and the object. The same finding has been reported for moisture removal of foodstuffs such as mint leaves (Ye et al., 2021) and thyme (Karami et al., 2021).

From the results obtained experimentally (Table 1), the ultrasonic pre-treatments showed good capability to reduce the required energy for the ginger slices dehydration where the longer sonication time, the lower the energy consumption. Mirzaei-Baktash et al. (2022) investigated the efficacy of sonication on energy consumption for mushroom drying in hot-air and electrohydrodynamic dryers; and reported that both the power level and application time caused noteworthy (*P < 0.05*) reductions in *SEC* in the dryers. Cao et al. (2018) evaluated freeze drying characteristics of barley grass; and confirmed that, in comparison with no pre-treatment, sonication at intensities of 10, 30 and 45 W/L allowed the consumed energy reduction of about 5, 8 and 19 %, respectively.

It is a worthy note that, according to the findings obtained through RSD validation (listed in Table 1), data of the specific energy consumption has generally good repeatability. In addition to the drying conditions and the object properties, temperature and relative humidity of the environmental wherein drying experiments are performed as well as the quality of the dryer components play important roles in the repeatability of the consumed energy data. Drying tests of the ginger samples were performed in a laboratory room with a precise control of environmental conditions.

### 3.3 Rehydration ratio

Rehydration ratio values for the dried gingers are represented in Table 2. As shown, the mean values for the ratio changed from 3.02 to 7.91 where the RSDs ranged from 5.84 to 23.87 %. Uniform prepared samples and steady controlled drying and rehydration conditions are essential to ensure good experimental repeatability for rehydration data. Based on the RSD values, it could be indicated that repeatability of the rehydration experiments has been relatively good.

**Table 2:** Rehydration ratio and total color change of gingers dried at the operating conditions.

T (°C)	V (m/s)	SD (min)	Rehydration ratio (-)		ΔE (-)	
			Mean	Relative standard deviation (%)	Mean	Relative standard deviation (%)
55	1	0	6.15	8.62	10.78	12.24
		15	7.47	10.58	10.05	8.46
		30	7.91	5.94	9.76	11.37
	2	0	5.33	17.07	10.44	11.88
		15	6.51	5.84	9.55	12.57
		30	7.17	6.28	9.73	8.12
	3	0	4.83	16.36	10.97	12.12
		15	5.52	18.30	9.46	8.56
		30	5.91	11.17	9.05	12.60
65	1	0	4.83	9.11	11.03	10.97
		15	6.73	10.85	10.48	6.11
		30	7.35	8.03	9.86	9.43
	2	0	4.81	20.17	11.86	4.64
		15	5.79	19.00	11.25	9.33
		30	6.23	13.96	10.46	7.55
	3	0	4.01	9.73	11.44	7.95
		15	5.07	23.87	10.49	6.48
		30	5.36	8.02	10.34	7.06
75	1	0	3.65	15.07	11.55	7.10
		15	4.92	14.84	10.19	6.77
		30	5.44	16.36	9.68	7.95
	2	0	3.43	12.54	10.56	10.89
		15	5.11	20.94	11.24	3.38
		30	5.65	14.34	10.43	6.14
	3	0	3.02	24.83	11.84	7.01
		15	3.97	16.12	10.09	9.61
		30	4.23	11.35	9.74	5.24

The capability of water absorption in cellular-structure products is a complicated phenomenon. In general, in addition to the innate characteristics of product, it is mainly governed by conditions of the experiments (drying and rehydration) (Hassan and Koca, 2022). According to the obtained results, the gingers dried at the higher temperatures had lower water absorption capability. The differences between temperature of 75 °C and the other practiced temperatures (65 and 55 °C) were found to be weighty (*P < 0.05*), where the mean rehydration ratios were determined to be 4.38, 5.58 and 6.31, respectively. The observation is may due to the increased surface hardening as well as shrinkage and damage of cells in the dried samples which resulting in decreased moisture permeability. The finding agrees well with the evidences in some research works conducted and reported. Izli and Polat (2019) investigated the rehydration capacity of ginger slices dried under convective and microwaves treatments, and stated that higher temperatures (in convective dryer) and power level (in microwave dryer) resulted in lower rehydration ratio of the samples.

For the air velocity, minor (*P > 0.05*) reduction in the rehydration ratio at the higher levels was observed which agrees well with the findings reported on tomato slices in a rotating-tray dryer (Santos-Sánchez et al., 2012) and on hot air dried apple slice (Vega-Gálvez et al., 2012). In general, because of the mechanism controls the mass transfer through the hot air drying, increasing the velocity makes the surface of the product harder and makes it much difficult for water penetration during the rehydration stage (Nozad et al., 2016).

As shown (Table 2), the sonication resulted in an increment in the rehydration ratio of the samples. From statistical analysis, practicing the sonication pre-treatment meaningfully (*P < 0.05*) influenced the rehydration capability in the dried gingers. The finding is in consistent with the observations of Bassey et al. (2022) for red dragon slices dried in an infrared dryer and Tüfekçi and Özkal (2017) for hot air dried okras. Based on the SEM micrographs derived by Tüfekçi and Özkal (2017) for okras pre-treated by ultrasonic cavitation, in addition to the cell walls of the samples, clear changes in collapses of full structure were also occurred. The phenomena is the main reason for the higher drying rates as well as the increased water absorption capability.

### 3.4 Total surface color change

Pigments’ destruction as well as browning reactions (enzymatic and non-enzymatic) on surface are the chief reasons govern the color change in the object (Bi et al., 2022). Natural pigments in foods have great potential to be changed and/or degraded under heat treatment; so that anthocyanins are converted to brown pigments and carotenoids isomerized to forms defined by a low-intensity color. According to the findings obtained through the experiments (Table 2) and conducted statistical analysis, in general, more total color change in the dried gingers’ surface was occurred at the higher drying air temperatures. This finding is confirmed by some researchers such as Beigi, 2019, Izli and Polat, 2019, Liu et al., 2021, and Bi et al. (2022). The main reason for the observation is due to the fact that reactivity between the sugar and amino group could be amplified by the increasing temperature and consequently the rate of Maillard reaction is enhanced (Aydogdu et al., 2015). Outcomes of the Maillard reaction are brown and known as a sequel of pigment formations.

From the results (Table 2), the impact of increasing air flow rate on the surface color change of the samples was not uniform and definite; so that in most cases led to a reduction while in some cases caused an increment in the parameter. However, based on statistical analysis, the influence of the velocity on the color change was not important (*P > 0.05*). The positive influence of increasing drying air velocity on the color retention is probably due to shorter the process duration (Bi et al., 2022). Mondal et al. (2022) reported the similar finding for maize grain drying in a mixed-flow dryer.

The average color change for sonication durations of 0, 15 and 30 min was obtained to be 11.33, 10.22 and 10.02, where the pre-treatment caused major (*P < 0.05*) reduction in the color deterioration in comparison with the untreated samples. However, the difference between the sonication duration of 15 and 30 min was not considerable (*P > 0.05*). Zhang et al. (2017) found that practicing sonication of dallies prior to drying in a hybrid microwave/convective dryer resulted in better color retention of the samples. Bozkir et al. (2019) used ultrasound pre-treatment for persimmon fruit, and assessed the quality properties of the dried samples. They confirmed that, compared to the untreated fruits, pre-treated samples with the ultrasonic power had low water activity and better color retention. Yue et al. (2023) assessed quality of *Codonopsis* slices after drying in a radio frequency vacuum dryer, and found that total surface color change of the pre-treated samples was slightly (*P > 0.05*) lower than for the non-treated slices. In contrast, Ren et al. (2022) used ultrasound at 40 kHz and 300 W for 15 min for Chinese ginger before drying in an infrared dryer, and observed noteworthy (*P < 0.05*) intensification in the surface color change in the samples treated by ultrasonic power (16.71 *vs.* 21.81).

Furthermore, relative standard deviation analysis revealed good repeatability for the color change data where the RSDs were determined to vary from 3.38 to 12.60 % (Table 2).

### 3.5 Main volatile component

According to the results obtained through the GC/MS analysis, the major volatile constituent for the fresh and dried gingers was identified to be α-Zingiberene. Concentration of the component in the fresh samples was 31.15 %. It is comparable with the findings reported for fresh ginger by Ding et al. (2012) (28.12 %) and Yamamoto-Ribeiro et al. (2013) (23.9 %).

The results for mean value of concentration of α-Zingiberene in the dried ginger slices as well as the RSD values is revealed in Table 3. Form the results, compared to the fresh gingers, drying process led to considerable (*P < 0.05*) augmentation in α-Zingiberene concentration. The observation is completely confirmed by Ding et al. (2012) who dried Chinese gingers using hot air (50, 60 and 70 °C), vacuum (13.3 kPa and 60 °C), freeze and microwave (60 W) drying methods.

**Table 3:** Extract yield (Y) and main volatile component (α-Zingiberene) of gingers dried at the operating conditions.

T (°C)	V (m/s)	SD (min)	Y (g/kg dry matter)		α-Zingiberene (%)	
			Mean	Relative standard deviation (%)	Mean	Relative standard deviation (%)
55	1	0	8.62	12.99	43.29	2.22
		15	9.92	8.57	44.32	2.66
		30	11.38	8.17	43.57	1.97
	2	0	9.35	7.91	43.16	3.38
		15	10.41	8.74	44.22	1.79
		30	11.74	9.20	43.97	2.66
	3	0	9.63	6.54	46.02	1.41
		15	10.74	6.98	45.86	2.88
		30	12.16	9.79	46.29	2.01
65	1	0	10.77	4.36	40.93	1.88
		15	12.73	9.51	42.38	2.41
		30	14.15	8.48	43.12	1.51
	2	0	10.43	8.05	41.29	2.35
		15	12.21	7.86	43.54	2.62
		30	13.38	3.81	42.97	3.23
	3	0	10.20	10.00	44.25	1.83
		15	11.41	5.87	46.19	1.15
		30	13.15	6.69	45.38	1.67
75	1	0	9.45	3.92	45.81	2.71
		15	11.49	7.92	46.38	2.09
		30	13.01	4.84	46.11	1.32
	2	0	9.13	7.01	41.45	2.17
		15	10.96	8.12	43.37	1.89
		30	11.45	11.44	44.75	2.41
	3	0	8.98	6.24	46.15	1.11
		15	10.37	8.97	46.59	1.70
		30	11.16	6.72	46.93	2.34

From the obtained results (Table 3), the average concentration for the of 55, 65 and 75 °C was 44.52, 43.34 and 45.28 % indicating that the highest temperature used to dry the gingers resulted in the maximum α-Zingiberene content. From statistical analysis, the differences among the temperatures were significant (*P < 0.05*). Average value of α-Zingiberene concentration was determined to be 43.99, 43.19 and 45.96 % for the flow rates of 1, 2 and 3 m/s, respectively. The results revealed that the highest drying air flow rate (3 m/s) resulted in an important (*P < 0.05*) increment in the concentration compared to the other levels (1 and 2 m/s). Furthermore, the average amount for the component concentration in the gingers dried without ultrasonic pre-treatment was 43.59 % while the value for samples dried after sonication for 15 and 30 min was 44.76 and 44.78 %, respectively.

From Table 3 and relying on the RSD results (1.11–3.38 %), it could be asserted that in addition to prepare uniform product and drying conditions, extraction process and the GC/MS analysis have been highly repeatable.

### 3.6 Extract yield

The average values for methanol extract of dried ginger slices with the related RSD values are listed in Table 3. RSD analysis indicated a good experimental repeatability for the extract yield data where the RSDs ranged from 3.81 to 12.99 % with a mean value of 7.73 %.

The yielded extract from the fresh samples was about 11.35 g/kg _dry matter_. The highest extract value (14.15 g/kg _dry matter_) was obtained from the gingers dried at 65 °C and 1 m/s after 30 min sonication. The lowest value (8.62 g/kg _dry matter_) yielded from the no-pre-treated samples and dried at 55 °C and 1 m/s.

From Table 3, the air temperature of 65 °C resulted to yield the highest amount (average of 12.05 g/kg _dry matter_) of the extract. The minimum average extract (10.44 g/kg _dry matter_) was yielded from the samples dried at 55 °C where the amount obtained at 75 °C was 10.73 g/kg _dry matter_. Based on statistical analysis, the difference between 55 °C and 75 °C was not important (*P > 0.05*).

From the results, at temperature of 55 °C and all the practiced sonication durations, increasing the air velocity resulted in a slight (*P > 0.05*) increment in the extract yield. However, for the other drying air temperatures (65 and 75 °C), a non-significant (*P > 0.05*) decrease was occurred following any increment in the velocity level.

The influence of the ultrasonic pre-treatment on the extract yield can also be discussed according to the results shown in Table 3. As revealed, at all the drying air temperatures and velocities practiced to dry the ginger slices, applying the pre-treatment and increasing its duration led to obtain more extract from the dried samples. As the statistical analysis revealed, in most cases, the influence was important (*P < 0.05*). The average extract yielded at the sonication durations of 0, 15 and 30 min was determined to be 9.62, 11.14 and 12.46 g/kg _dry matter_, respectively.

### 3.7 TPC and antioxidant activity

Determined total phenolic content (TPC) and antioxidant ativity of the methanol extract of the dried gingers and the related RSDs are listed in Table 4. The ranges for TPC (16.86–19.63 mg GAE/g dry matter) and antioxidant activity (71.32–88.95 %) are comparable with the reported results for dried ginger by some researchers such as Thuwapanichayanan et al. (2014) (16.90–22.73 mg GAE/g dry matter and 87.25–90.59 %), Gümüşay et al. (2015) (2.84–9.10 mg GAE/g dry matter) and Mustafa and Chin (2023) (8.48–9.09 GAE/g dry matter).

**Table 4:** Total phenolic content (TPC) and antioxidant activity (AA) of methanol extract of gingers dried at the operating conditions.

T (°C)	V (m/s)	SD (min)	TPC (mg GAE/g dry matter)		AA (%)	
			Mean	Relative standard deviation (%)	Mean	Relative standard deviation (%)
55	1	0	18.09	6.47	77.83	3.58
		15	17.26	4.87	73.33	2.22
		30	16.86	7.65	71.32	2.78
	2	0	18.59	3.39	80.94	2.63
		15	17.87	4.20	76.57	2.01
		30	17.31	5.08	73.59	1.18
	3	0	18.83	5.47	82.59	1.77
		15	18.07	3.98	77.72	2.51
		30	17.59	5.51	75.05	1.11
65	1	0	19.17	4.80	85.11	0.86
		15	18.26	1.97	78.85	2.27
		30	17.54	3.36	74.78	2.07
	2	0	19.58	5.26	88.51	1.20
		15	18.63	4.03	81.21	0.57
		30	17.94	2.45	76.97	1.04
	3	0	19.63	4.13	88.95	1.59
		15	18.82	5.05	82.52	1.60
		30	18.06	3.49	77.66	2.59
75	1	0	18.76	1.65	82.10	2.29
		15	18.24	6.41	78.73	1.23
		30	17.38	4.37	73.95	1.77
	2	0	18.59	2.85	81.21	1.81
		15	17.68	4.58	75.53	2.05
		30	16.97	6.31	71.87	1.57
	3	0	19.15	4.65	84.96	1.01
		15	18.53	3.99	80.55	1.54
		30	17.78	3.88	76.08	2.20

The average TPC and antioxidant activity for 55, 65 and 75 °C was calculated to be 17.83, 18.62 and 18.12 GAE/g dry matter and 76.55, 81.61 and 78.33 %, respectively indicating that the temperature has importantly (*P < 0.05*) affected both the parameters. This finding is completely supported by the observations of Thuwapanichayanan et al. (2014) and Zeng et al. (2023).

The average of both the TPC and antioxidant activity of the extract from the dried gingers was slightly (*P > 0.05*) increased following increasing the velocity. The mean values for TPC and antioxidant activity for sonication duration of 0, 5 and 30 min was determined to be 18.93, 18.15 and 17.49 GAE/g dry matter and 83.57, 78.33 and 74.58 %, respectively. Based on the conducted statistical analysis, increasing ultrasonic pre-treatment time meaningfully (*P < 0.05*) resulted in reduced both the TPC and antioxidant activity of the dried gingers.

Furthermore, for both the TPC and antioxidant activity, analysis of RSD indicated great repeatability for the experimental data.

### 3.8 Multi-objective optimization

**Fig. 1:**
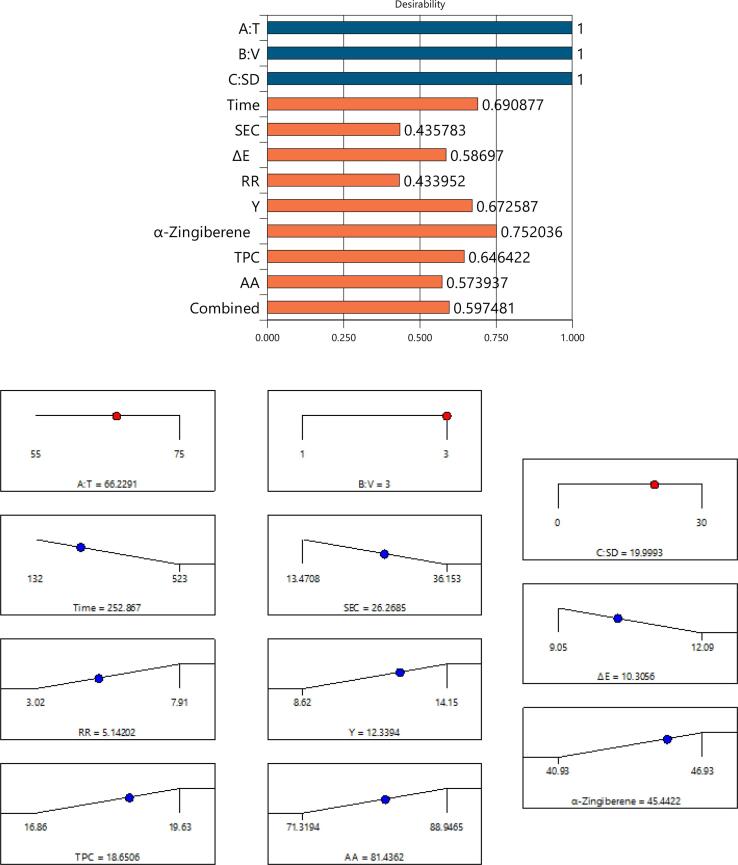
Results obtained for individual desirability values for the different studies parameters of ginger slices.

**Fig. 2:**
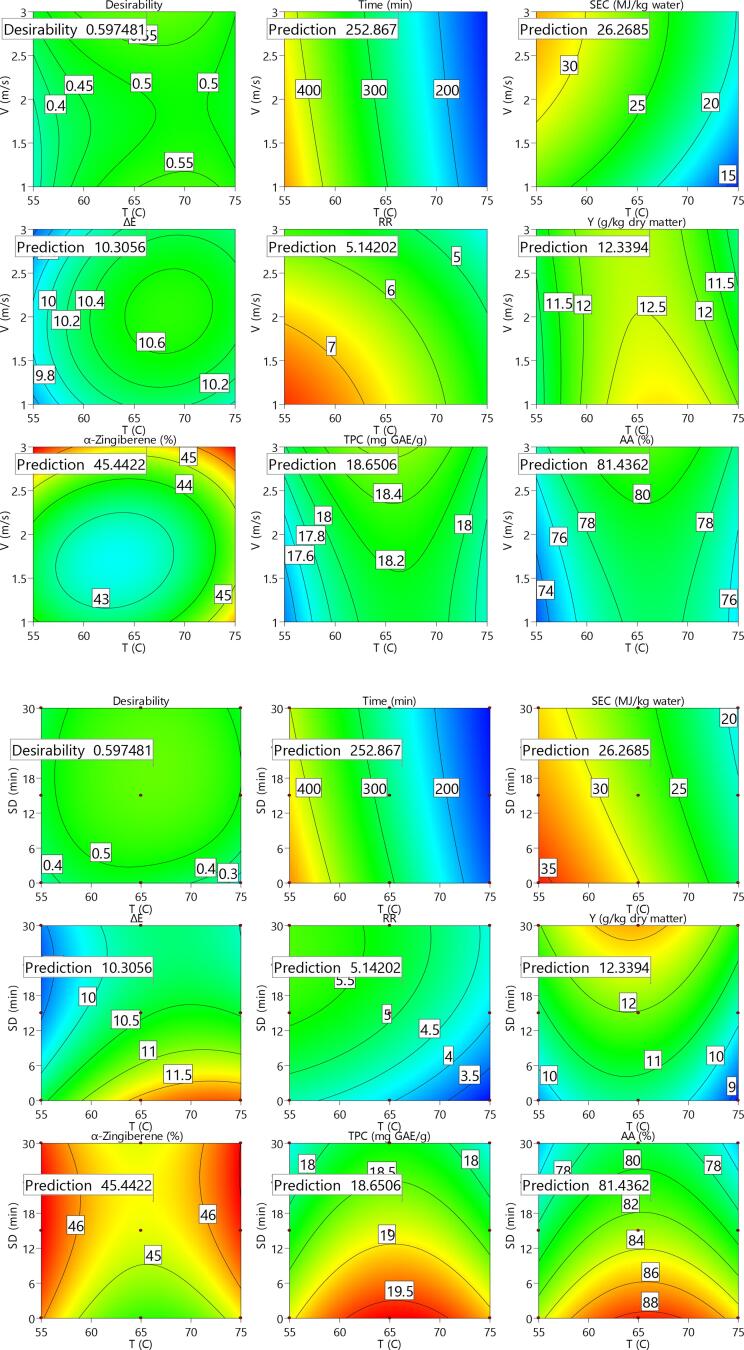

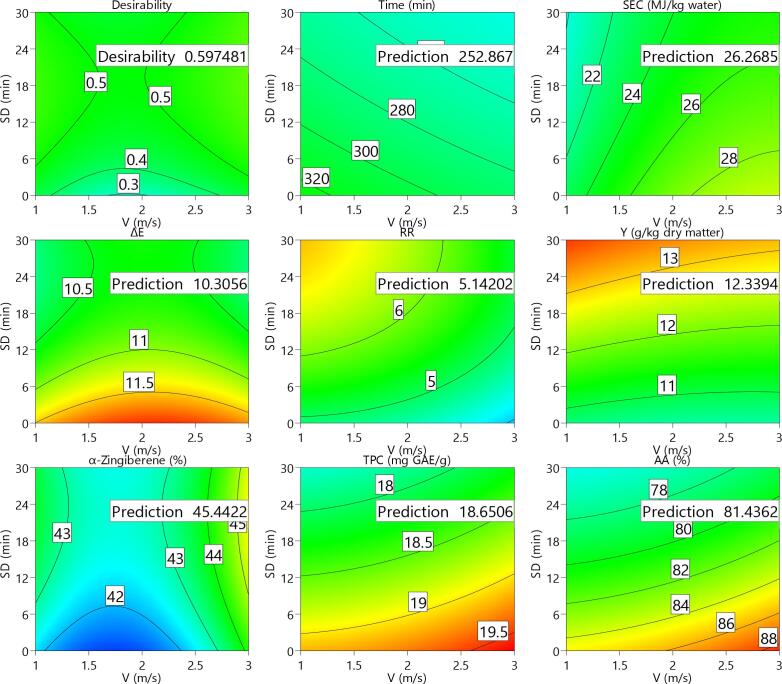
Response surface and contours of the studied parameters of ginger slices.

The values obtained for individual desirability of the studied control factors and response variable as well as the combined optimization for ginger slices are shown in Fig. 1. Furthermore, Fig. 2 represents response surface and contours of the studied parameters of the ginger slices. As re revealed, the desirability for the temperature (T), velocity (V) and sonication duration (SD) is equal to unit as the values are in the practiced range during the experiments. The combined desirability was determined to be about 0.597 which is relatively low. This is mainly due to the fact that, in addition to opposite effects on the evaluated indicators, each of the studied drying parameters for a specific trait did not showed a constant trend in the most cases. The desired independent factors were revealed to be about 66 °C temperature, 3 m/s velocity and 20 min sonication duration. Under such drying conditions, the optimum responses for drying duration, *SEC*, rehydration ratio, change in the surface color, yielded extract, main chemical composition, TPC, and antioxidant activity were predicted to be about 252.3 min, 26.27 MJ/kg, 5.14, 10.31, 12.33 (g/kg dry matter), 45.44 (%), 18.65 (GAE/g dry matter) and 81.43 %, respectively.

## 4 Conclusion

The usefulness of sonication before forced hot air processing on dehydration features of ginger slices was investigated. According to the observations obtained through highly repeatable experiments, the sonication prior to the drying process had several considerable advantages including reduced the consumed time and energy as well as higher rehydration capacity, less color degradation and more extract yield of the dried slices. However, in general, the ultrasonic pre-treatment resulted in lower both the total phenolic content and antioxidant activity of the ginger slices. Response surface methodology revealed 20 min sonication pre-treatment and drying at 66 °C and of 3 m/s as the most desirable conditions for dehydration of the ginger slices.

## Funding

This research was funded by the National Key Research and Development Program of China Sub-project grant number [No. 2021YFD2000705].

## CRediT authorship contribution statement

**Kaikang Chen:** Conceptualization. **Yanwei Yuan:** Methodology. **Bo Zhao:** Writing – review & editing. **Mohammad Kaveh:** Formal analysis. **Mohsen Beigi:** Writing – review & editing. **Yongjun Zheng:** Writing – original draft. **Mehdi Torki:** Writing – review & editing.

## Declaration of Competing Interest

The authors declare that they have no known competing financial interests or personal relationships that could have appeared to influence the work reported in this paper.

## Data Availability

Data will be made available on request.
